# Supplementary material for: Functional Profiling of DNA Repair Pathways in Lung Cancer Patients Uncovers Radiotherapy-Induced and Cancer-Associated Alterations in Oxidative Lesion Repair
Source: medRxiv. 2026 Jan 13:2026.01.12.26343971. Preprint. [Version 1] doi: 10.64898/2026.01.12.26343971 (PMC12870641; doi:10.64898/2026.01.12.26343971)
Supplement: Supplement 1 [file NIHPP2026.01.12.26343971v1-supplement-1.pdf]

medRxiv preprint doi: https://doi.org/10.64898/2026.01.12.26343971; this version posted January 13, 2026. The copyright holder for this preprint (which was not certified by peer review) is the author/funder, who has granted medRxiv a license to display the preprint in perpetuity.
It is made available under a CC-BY 4.0 International license .

Supplementary table 1. Crude association between baseline DNA repair capacity and patient characteristics.

Supplementary table 2. Radiotherapy response in DNA repair capacity and by patient status.

Supplementary table 3. Correlations of DNA repair capacity measurements at baseline and at post-radiotherapy visit.

Supplementary table 4. Radiotherapy response in DNA repair capacity by follow-up months.

Supplementary table 5. Case control comparison for DNA repair capacity.

medRxiv preprint doi: https://doi.org/10.64898/2026.01.12.26343971; this version posted January 13, 2026. The copyright holder for this preprint (which was not certified by peer review) is the author/funder, who has granted medRxiv a license to display the preprint in perpetuity.
It is made available under a CC-BY 4.0 International license .

Supplementary figure 1: Hierarchy of cell selection in flow cytometry

medRxiv preprint doi: https://doi.org/10.64898/2026.01.12.26343971; this version posted January 13, 2026. The copyright holder for this preprint (which was not certified by peer review) is the author/funder, who has granted medRxiv a license to display the preprint in perpetuity.
It is made available under a CC-BY 4.0 International license .

(A) Scatter plots illustrating gates used for identifying singlets using flow cytometry in forward scatter (FSC) versus side scatter (SSC).
(B) Gating hierarchy.

Supplementary figure 2: Representative flow cytometry scatter plots for PBMCs transfected with the reporter plasmid cocktails described in Table 1.

Supplementary figure 3: Cancer type distribution (A) and associated baseline DNA repair capacity variations (B).

Supplementary figure 4: Baseline DNA repair capacity by demographics. Z-scored DNA repair capacity (DRC) at the pre-radiotherapy (baseline) timepoint is evaluated in relation to patient demographics: **(A)** age (in years), **(B)** sex (male, female) and **(C)** smoking status (never, former, current).

Supplementary figure 5: Radiotherapy-induced changes in DNA repair capacity by demographics. Changes in DRC (post-RT minus baseline, z-scored) are assessed in relation to: **(A)** age (in years), **(B)** sex (male, female) and **(C)** smoking status (never, former, current).

Supplementary figure 6: Correlation and paired comparisons of DNA repair capacity across timepoints in patient samples.
(A) Correlations between pre-RT and post-RT samples within the same patients.
(B) Correlations between two independent post-RT samples collected from the same patients at distinct follow-up timepoints.
(C) Paired comparisons of DNA repair capacity across pre-RT, post-RT, and additional post-RT visits (PostNew) within the same patients. Statistical significance of pairwise comparisons is indicated by p-values derived from linear mixed-effects models.

Supplementary figure 7. Technical reproducibility and assay validation.
(A) Reproducibility of DRC assays assessed in a subset of patients (IDs 1-5) with pre-RT (baseline) samples measured in two separate years: DRC measurements (z-scores) at 2023 (Replicate 1) and 2024 (Replicate 2). Each point represents one measurement per replicate, colored by assay type.
(B) Per-assay reproducibility across NHEJ, LP-BER, MMR, U:G, 8oxoG:C, A:8oxoG, IR(3000Gy), Hx(MPG), and NER, was assessed in replicate samples from case individuals. Replicates were processed in two different years: 2023 (Replicate 1) and 2024 (Replicate 2). Each panel shows z-scored DRC values for the two replicates, along with Spearman correlation coefficients and significance (ns = not significant).
(C) Spearman correlation between DRC and PBMC cell count across collected samples.

Supplementary table 1. Crude association between baseline DNA repair capacity and patient characteristics.

	NHEJ			LP-PBER			MMR			U:G		
**Patient characteristics**	beta	SE	p_value	beta	SE	p_value	beta	SE	p_value	beta	SE	p_value
Age (years)	-0.002	0.007	0.782	0.003	0.009	0.741	-0.002	0.007	0.780	-0.002	0.006	0.701
Sex: femae vs male	0.035	0.135	0.797	0.190	0.174	0.277	0.120	0.130	0.362	-0.016	0.121	0.895
Smoking: former vs never	0.080	0.169	0.637	-0.140	0.217	0.521	0.061	0.165	0.713	0.058	0.151	0.704
Smoking: current vs never	0.120	0.267	0.655	-0.387	0.343	0.261	-0.051	0.260	0.844	0.020	0.246	0.934
Primary diagnosis: non-nsclc vs nsclc	0.130	0.144	0.367	-0.086	0.187	0.649	-0.001	0.140	0.992	0.024	0.129	0.856
Cancer stage: locally advanced vs early stage	**0.441**	**0.182**	**0.017**	0.038	0.249	0.879	-0.260	0.182	0.156	0.093	0.172	0.590
Cancer stage: oligomet vs early stage	0.036	0.196	0.854	-0.024	0.268	0.928	**-0.378**	**0.195**	**0.056**	-0.027	0.182	0.883
Cancer stage: widely met vs early stage	0.218	0.273	0.427	0.018	0.373	0.962	-0.237	0.272	0.385	0.302	0.252	0.234
Cancer stage: trend test	-0.024	0.079	0.764	-0.010	0.103	0.922	-0.111	0.076	0.147	0.036	0.071	0.613
Number of primary diagnosis	0.027	0.109	0.809	-0.151	0.140	0.284	-0.161	0.117	0.173	0.090	0.103	0.388
Type: radical vs sbrt	0.160	0.159	0.319	-0.072	0.208	0.731	0.063	0.153	0.680	0.099	0.142	0.489
Type: palliative vs sbrt	-0.039	0.327	0.905	0.157	0.426	0.714	**0.582**	**0.312**	**0.066**	0.205	0.288	0.479
Prior radiotherapy (any)	**-0.367**	**0.182**	**0.047**	-0.356	0.241	0.143	-0.094	0.181	0.604	-0.074	0.165	0.656
Prior radiotherapy (within 6 months)	**-0.533**	**0.274**	**0.055**	-0.569	0.360	0.118	0.161	0.271	0.553	-0.205	0.246	0.406
Prior chemotherapy	0.109	0.145	0.455	0.117	0.189	0.538	0.113	0.141	0.426	0.174	0.129	0.181
Prior immunotherapy	-0.102	0.197	0.606	0.150	0.256	0.559	-0.071	0.191	0.711	-0.153	0.174	0.381
Prior targeted therapy	-0.114	0.181	0.531	0.200	0.235	0.396	-0.204	0.174	0.245	0.150	0.159	0.349
Race: black vs white	-0.328	0.470	0.487	0.167	0.619	0.788	**0.972**	**0.455**	**0.035**	0.323	0.422	0.445
Race: asian vs white	-0.334	0.386	0.388	0.044	0.508	0.930	-0.125	0.373	0.739	0.145	0.346	0.676

	8oxoG:C			A:8oxoG			IR(3000Gy)			Hx(MPG)			NER		
**Patient characteristics**	beta	SE	p_value	beta	SE	p_value	beta	SE	p_value	beta	SE	p_value	beta	SE	p_value
Age (years)	-0.015	0.009	0.108	0.001	0.009	0.899	0.000	0.009	0.977	-0.010	0.007	0.198	0.000	0.009	0.974
Sex: femae vs male	-0.131	0.186	0.483	0.208	0.175	0.236	0.141	0.169	0.404	-0.203	0.143	0.159	0.233	0.168	0.170
Smoking: former vs never	0.112	0.230	0.626	0.211	0.216	0.331	0.038	0.213	0.857	0.093	0.182	0.609	0.155	0.215	0.471
Smoking: current vs never	-0.394	0.394	0.320	0.320	0.340	0.350	-0.113	0.349	0.747	-0.015	0.287	0.959	0.244	0.338	0.472
Primary diagnosis: non-nsclc vs nsclc	0.262	0.199	0.192	-0.076	0.188	0.687	0.043	0.182	0.813	-0.059	0.155	0.702	-0.057	0.182	0.756
Cancer stage: locally advanced vs early stage	0.146	0.269	0.590	0.368	0.245	0.137	**-0.490**	**0.232**	**0.037**	-0.285	0.202	0.161	-0.312	0.237	0.192
Cancer stage: oligomet vs early stage	-0.179	0.287	0.534	0.099	0.263	0.708	-0.409	0.249	0.104	-0.118	0.217	0.589	-0.419	0.256	0.105
Cancer stage: widely met vs early stage	0.045	0.410	0.913	0.205	0.367	0.577	**-0.796**	**0.347**	**0.024**	0.151	0.303	0.620	-0.484	0.356	0.178
Cancer stage: trend test	-0.076	0.113	0.501	0.005	0.103	0.960	**-0.191**	**0.097**	**0.053**	0.046	0.085	0.592	-0.163	0.099	0.103
Number of primary diagnosis	0.107	0.162	0.512	-0.185	0.132	0.167	0.047	0.133	0.725	**-0.201**	**0.114**	**0.081**	-0.032	0.136	0.812
Type: radical vs sbrt	0.134	0.219	0.543	0.273	0.207	0.190	0.036	0.201	0.859	-0.170	0.169	0.318	-0.002	0.202	0.993
Type: palliative vs sbrt	0.274	0.443	0.538	0.312	0.423	0.462	-0.078	0.411	0.850	0.376	0.347	0.282	0.360	0.413	0.386
Prior radiotherapy (any)	0.192	0.254	0.451	-0.304	0.241	0.210	-0.208	0.233	0.376	0.209	0.200	0.300	-0.051	0.235	0.827
Prior radiotherapy (within 6 months)	-0.204	0.380	0.592	**-0.664**	**0.358**	**0.067**	0.130	0.351	0.711	0.302	0.301	0.318	0.191	0.351	0.587
Prior chemotherapy	0.196	0.202	0.336	0.158	0.189	0.405	-0.206	0.183	0.263	**0.312**	**0.153**	**0.044**	-0.013	0.184	0.943
Prior immunotherapy	-0.200	0.268	0.458	-0.073	0.257	0.776	0.043	0.247	0.863	**0.483**	**0.207**	**0.022**	0.103	0.249	0.679
Prior targeted therapy	-0.028	0.253	0.913	0.286	0.234	0.225	0.087	0.227	0.701	-0.207	0.194	0.288	-0.158	0.228	0.491
Race: black vs white	-0.611	0.640	0.342	-0.215	0.629	0.734	0.740	0.584	0.208	**0.842**	**0.503**	**0.098**	0.669	0.598	0.266
Race: asian vs white	-0.471	0.526	0.373	0.305	0.517	0.556	0.300	0.480	0.534	-0.112	0.413	0.787	-0.477	0.491	0.333

Statistics are obtained from univariate linear regression models.

Abbreviations: SE = standard error.

Supplementary table 2. Radiotherapy response in DNA repair capacity and by patient status.

	All patients (N=100)								
**Model**	**a**			**b**			**c**		
**Response pathways**	beta	SE	p_value	beta	SE	p_value	beta	SE	p_value
NHEJ	0.026	0.080	0.742	0.028	0.080	0.726	0.031	0.081	0.701
LP-BER	0.159	0.095	0.098	0.161	0.095	0.093	0.156	0.097	0.113
MMR	-0.036	0.064	0.577	-0.037	0.064	0.571	-0.032	0.066	0.627
U:G	**-0.170**	**0.075**	**0.025**	**-0.167**	**0.075**	**0.028**	**-0.170**	**0.077**	**0.029**
8oxoG:C	0.109	0.079	0.175	0.107	0.080	0.180	0.098	0.081	0.228
A:8oxoG	**-0.175**	**0.083**	**0.038**	**-0.175**	**0.083**	**0.038**	**-0.200**	**0.084**	**0.020**
IR(3000Gy)	-0.026	0.089	0.775	-0.028	0.089	0.756	-0.027	0.091	0.765
Hx(MPG)	0.000	0.069	0.997	-0.001	0.069	0.983	0.007	0.070	0.918
NER	0.005	0.075	0.943	0.006	0.075	0.939	0.018	0.076	0.816

a. crude model: no adjustment
b. adjusted for cancer stage and RT type
c. fully adjusted model: age, sex, smoking, cancer stage, histology, RT type, dosage, fraction, prior RT history, prior chemotherapy, prior immunotherapy, prior targeted therapy, during-RT chemotherapy, during-RT immunotherapy, during-RT targeted therapy.
Statistics are obtained from linear mixed effects models with radiotherapy status as the main effect variable.

	**Non-NSCLC (N=32)**			**NSCLC (N=68)**		
**Response pathways**	beta	SE	p_value	beta	SE	p_value
NHEJ	0.053	0.129	0.685	0.012	0.101	0.904
LP-BER	0.215	0.169	0.211	0.130	0.117	0.270
MMR	0.113	0.109	0.305	-0.108	0.079	0.176
U:G	-0.130	0.145	0.378	**-0.187**	**0.086**	**0.034**
8oxoG:C	0.253	0.151	0.105	0.045	0.092	0.627
A:8oxoG	-0.128	0.172	0.464	**-0.196**	**0.093**	**0.038**
IR(3000Gy)	0.090	0.166	0.591	-0.081	0.105	0.446
Hx(MPG)	0.089	0.127	0.490	-0.040	0.083	0.627
NER	0.089	0.142	0.536	-0.041	0.087	0.641

**Early stage (N=18)**			**Locally advanced (N=45)**			**Oligomet (N=29)**			**Widely met (N=8)**		
beta	SE	p_value	beta	SE	p_value	beta	SE	p_value	beta	SE	p_value
0.057	0.186	0.761	-0.060	0.125	0.633	0.241	0.136	0.087	-0.295	0.229	0.239
0.088	0.213	0.684	0.206	0.151	0.180	0.159	0.190	0.410	0.068	0.154	0.671
-0.161	0.162	0.337	-0.062	0.085	0.466	0.071	0.135	0.606	0.013	0.239	0.959
-0.235	0.197	0.241	-0.091	0.118	0.443	-0.137	0.117	0.252	**-0.593**	**0.240**	**0.043**
0.311	0.193	0.128	0.022	0.133	0.872	0.082	0.128	0.530	0.237	0.181	0.240
0.106	0.263	0.693	**-0.327**	**0.116**	**0.008**	-0.073	0.120	0.547	-0.380	0.293	0.216
-0.286	0.191	0.154	-0.018	0.134	0.894	-0.004	0.175	0.981	0.370	0.265	0.206
-0.233	0.141	0.119	0.070	0.119	0.561	0.120	0.102	0.249	-0.229	0.232	0.363
-0.191	0.129	0.158	0.073	0.114	0.528	0.047	0.144	0.746	-0.055	0.338	0.874

	**All patients (N=100)**								
	**SBRT (N=24)**			**Radical (N=71)**			**Palliative (N=5)**		
**Response pathways**	beta	SE	p_value	beta	SE	p_value	beta	SE	p_value
NHEJ	0.027	0.165	0.870	0.030	0.096	0.754	-0.078	0.310	0.813
LP-BER	0.155	0.231	0.508	0.161	0.110	0.146	0.074	0.199	0.728
MMR	0.070	0.168	0.680	-0.060	0.070	0.391	-0.153	0.281	0.615
U:G	-0.088	0.114	0.445	-0.159	0.095	0.100	*-0.736*	*0.280*	*0.058*
8oxoG:C	-0.062	0.112	0.583	0.169	0.102	0.102	-0.092	0.344	0.803
A:8oxoG	-0.051	0.139	0.719	*-0.203*	*0.106*	*0.059*	-0.427	0.308	0.239
IR(3000Gy)	0.013	0.158	0.933	-0.048	0.112	0.669	0.114	0.327	0.745
Hx(MPG)	0.065	0.122	0.599	0.004	0.085	0.967	-0.276	0.345	0.469
NER	0.005	0.121	0.967	0.039	0.094	0.676	-0.408	0.350	0.308

**Non-NSCLC (N=32)**								
**SBRT (N=11)**			**Radical (N=18)**			**Palliative (N=3)**		
beta	SE	p_value	beta	SE	p_value	beta	SE	p_value
-0.101	0.195	0.615	0.131	0.189	0.499	0.017	0.455	0.973
-0.205	0.257	0.441	0.469	0.242	0.070	0.257	0.242	0.400
0.408	0.203	0.071	0.023	0.121	0.849	-0.373	0.388	0.438
-0.125	0.169	0.475	-0.063	0.238	0.794	-0.637	0.343	0.205
-0.192	0.158	0.256	**0.506**	**0.228**	**0.042**	0.137	0.422	0.777
-0.048	0.246	0.850	-0.110	0.264	0.682	-0.815	0.355	0.148
-0.268	0.240	0.287	0.309	0.238	0.214	0.173	0.563	0.788
0.300	0.204	0.171	0.076	0.154	0.629	-0.539	0.556	0.435
-0.109	0.209	0.612	0.242	0.213	0.276	-0.072	0.354	0.858

**NSCLC (N=68)**								
**SBRT (N=13)**			**Radical (N=53)**			**Palliative (N=2)**		
beta	SE	p_value	beta	SE	p_value	beta	SE	p_value
0.127	0.265	0.641	-0.005	0.113	0.963	-0.222	0.412	0.644
0.481	0.336	0.167	0.058	0.120	0.633	-0.200	0.310	0.635
-0.235	0.230	0.328	-0.090	0.085	0.291	0.178	0.276	0.586
-0.051	0.159	0.756	*-0.190*	*0.101*	*0.065*	-0.886	0.520	0.231
0.038	0.157	0.812	0.064	0.110	0.566	-0.434	0.515	0.488
-0.041	0.149	0.788	**-0.243**	**0.112**	**0.034**	0.156	0.081	0.303
0.274	0.187	0.171	-0.162	0.124	0.196	0.025	0.324	0.950
-0.132	0.112	0.266	-0.019	0.102	0.853	0.118	0.076	0.365
0.103	0.131	0.448	-0.035	0.102	0.734	-0.912	0.651	0.394

	**Dosage<=60Gy (N=82)**			**Dosage>60Gy (N=18)**		
**Response pathways**	beta	SE	p_value	beta	SE	p_value
NHEJ	0.057	0.089	0.523	-0.118	0.181	0.524
LP-BER	**0.258**	**0.108**	**0.019**	-0.313	0.161	0.070
MMR	-0.073	0.068	0.288	0.132	0.179	0.472
U:G	-0.168	0.083	0.047	-0.185	0.179	0.319
8oxoG:C	**0.180**	**0.084**	**0.034**	-0.265	0.212	0.232
A:8oxoG	-0.100	0.093	0.285	**-0.518**	**0.169**	**0.008**
IR(3000Gy)	0.037	0.097	0.706	-0.299	0.217	0.186
Hx(MPG)	-0.022	0.073	0.764	0.090	0.198	0.657
NER	0.007	0.085	0.933	-0.009	0.158	0.953

**Fraction<=20 (N=41)**			**Fraction>20 (N=59)**		
beta	SE	p_value	beta	SE	p_value
0.083	0.109	0.450	-0.010	0.113	0.932
0.235	0.145	0.114	0.104	0.127	0.416
0.034	0.102	0.741	-0.086	0.083	0.307
-0.126	0.101	0.219	-0.208	0.108	0.060
0.152	0.106	0.159	0.078	0.115	0.500
0.035	0.113	0.756	**-0.334**	**0.114**	**0.005**
0.084	0.139	0.546	-0.107	0.116	0.362
-0.077	0.103	0.459	0.057	0.093	0.545
-0.008	0.124	0.947	0.017	0.093	0.858

Supplementary table 3. Correlations of DNA repair capacity measurements at baseline and at post-radiotherapy visit.

Baseline Pearson's correlation coefficient

	NHEJ	LP-BER	MMR	U:G	8oxoG:C	A:8oxoG	IR(3000Gy)	Hx(MPG)	NER
NHEJ	1.000	0.499	0.089	-0.106	0.165	0.017	0.089	0.062	0.087
LP-BER	0.499	1.000	0.424	0.019	0.052	0.223	0.463	-0.024	0.367
MMR	0.089	0.424	1.000	-0.077	-0.071	0.165	0.303	0.368	0.332
U:G	-0.106	0.019	-0.077	1.000	0.119	0.073	0.123	-0.123	0.120
8oxoG:C	0.165	0.052	-0.071	0.119	1.000	0.135	0.035	0.043	0.045
A:8oxoG	0.017	0.223	0.165	0.073	0.135	1.000	0.097	-0.136	0.007
IR(3000Gy)	0.089	0.463	0.303	0.123	0.035	0.097	1.000	-0.082	0.794
Hx(MPG)	0.062	-0.024	0.368	-0.123	0.043	-0.136	-0.082	1.000	-0.014
NER	0.087	0.367	0.332	0.120	0.045	0.007	0.794	-0.014	1.000

Post-RT Pearson's correlation coefficient

	NHEJ	LP-BER	MMR	U:G	8oxoG:C	A:8oxoG	IR(3000Gy)	Hx(MPG)	NER
NHEJ	1.000	0.394	-0.159	-0.124	0.097	-0.167	-0.077	0.064	0.009
LP-BER	0.394	1.000	0.128	0.020	0.024	0.261	0.348	-0.069	0.376
MMR	-0.159	0.128	1.000	-0.013	0.168	0.211	0.394	0.288	0.320
U:G	-0.124	0.020	-0.013	1.000	0.202	0.263	0.160	0.001	0.131
8oxoG:C	0.097	0.024	0.168	0.202	1.000	0.159	-0.027	0.277	0.003
A:8oxoG	-0.167	0.261	0.211	0.263	0.159	1.000	0.150	-0.014	0.057
IR(3000Gy)	-0.077	0.348	0.394	0.160	-0.027	0.150	1.000	-0.201	0.772
Hx(MPG)	0.064	-0.069	0.288	0.001	0.277	-0.014	-0.201	1.000	-0.177
NER	0.009	0.376	0.320	0.131	0.003	0.057	0.772	-0.177	1.000

Baseline Pearson's correlation p value (raw on the lower diagnol and multiple-testing adjusted on the upper)

	NHEJ	LP-BER	MMR	U:G	8oxoG:C	A:8oxoG	IR(3000Gy)	Hx(MPG)	NER
NHEJ	0.000	0.000	1.000	1.000	1.000	1.000	1.000	1.000	1.000
LP-BER	0.000	0.000	0.001	1.000	1.000	0.807	0.000	1.000	0.007
MMR	0.391	0.000	0.000	1.000	1.000	1.000	0.082	0.007	0.028
U:G	0.310	0.860	0.462	0.000	1.000	1.000	1.000	1.000	1.000
8oxoG:C	0.117	0.626	0.503	0.263	0.000	1.000	1.000	1.000	1.000
A:8oxoG	0.868	0.029	0.109	0.487	0.199	0.000	1.000	1.000	1.000
IR(3000Gy)	0.388	0.000	0.003	0.238	0.739	0.348	0.000	1.000	0.000
Hx(MPG)	0.549	0.818	0.000	0.241	0.683	0.185	0.425	0.000	1.000
NER	0.398	0.000	0.001	0.252	0.668	0.950	0.000	0.892	0.000

Post-RT Pearson's correlation p value (raw on the lower diagnol and multiple-testing adjusted on the upper)

	NHEJ	LP-BER	MMR	U:G	8oxoG:C	A:8oxoG	IR(3000Gy)	Hx(MPG)	NER
NHEJ	0.000	0.002	1.000	1.000	1.000	1.000	1.000	1.000	1.000
LP-BER	0.000	0.000	1.000	1.000	1.000	0.305	0.019	1.000	0.006
MMR	0.124	0.215	0.000	1.000	1.000	1.000	0.003	0.161	0.054
U:G	0.238	0.847	0.905	0.000	1.000	0.305	1.000	1.000	1.000
8oxoG:C	0.363	0.819	0.113	0.055	0.000	1.000	1.000	0.228	1.000
A:8oxoG	0.107	0.011	0.042	0.011	0.132	0.000	1.000	1.000	1.000
IR(3000Gy)	0.462	0.001	0.000	0.126	0.803	0.150	0.000	1.000	0.000
Hx(MPG)	0.543	0.510	0.005	0.992	0.008	0.893	0.054	0.000	1.000
NER	0.935	0.000	0.002	0.211	0.977	0.587	0.000	0.090	0.000

RT-change Pearson's correlation coefficient

	NHEJ	LP-BER	MMR	U:G	8oxoG:C	A:8oxoG	IR(3000Gy)	Hx(MPG)	NER
NHEJ	1.000	0.535	-0.138	0.087	0.234	0.024	0.024	0.054	0.008
LP-BER	0.535	1.000	-0.239	-0.049	0.189	0.245	0.297	-0.065	0.247
MMR	-0.138	-0.239	1.000	0.098	0.039	-0.055	0.035	0.350	0.105
U:G	0.087	-0.049	0.098	1.000	0.194	0.326	0.120	0.071	0.220
8oxoG:C	0.234	0.189	0.039	0.194	1.000	0.298	0.178	0.013	0.196
A:8oxoG	0.024	0.245	-0.055	0.326	0.298	1.000	0.131	-0.022	0.079
IR(3000Gy)	0.024	0.297	0.035	0.120	0.178	0.131	1.000	-0.146	0.728
Hx(MPG)	0.054	-0.065	0.350	0.071	0.013	-0.022	-0.146	1.000	-0.088
NER	0.008	0.247	0.105	0.220	0.196	0.079	0.728	-0.088	1.000

Baseline (row) - Post-RT (column) Perason's correlation coefficient

	NHEJ	LP-BER	MMR	U:G	8oxoG:C	A:8oxoG	IR(3000Gy)	Hx(MPG)	NER
NHEJ	0.402	0.115	0.089	-0.123	0.036	-0.156	0.099	0.021	0.249
LP-BER	0.081	0.374	0.493	0.047	0.036	0.126	0.385	0.059	0.478
MMR	-0.060	0.240	0.628	-0.246	-0.015	0.133	0.369	0.016	0.282
U:G	-0.205	0.124	-0.001	0.345	0.127	-0.062	0.178	0.139	0.069
8oxoG:C	0.040	-0.105	0.073	0.035	0.651	-0.081	-0.088	0.220	-0.031
A:8oxoG	0.008	0.167	0.247	0.086	0.151	0.511	0.130	0.022	0.010
IR(3000Gy)	-0.118	0.076	0.276	-0.020	-0.060	-0.038	0.427	-0.026	0.462
Hx(MPG)	0.063	-0.101	0.313	-0.283	0.102	-0.143	-0.087	0.526	-0.126
NER	-0.028	0.179	0.280	0.002	-0.077	-0.077	0.432	0.034	0.578

RT-change Pearson's correlation p value (raw on the lower diagnol and multiple-testing adjusted on the upper)

	NHEJ	LP-BER	MMR	U:G	8oxoG:C	A:8oxoG	IR(3000Gy)	Hx(MPG)	NER
NHEJ	0.000	0.000	1.000	1.000	0.766	1.000	1.000	1.000	1.000
LP-BER	0.000	0.000	0.590	1.000	1.000	0.542	0.129	1.000	0.522
MMR	0.188	0.021	0.000	1.000	1.000	1.000	1.000	0.024	1.000
U:G	0.415	0.647	0.364	0.000	1.000	0.060	1.000	1.000	0.993
8oxoG:C	0.028	0.078	0.722	0.071	0.000	0.150	1.000	1.000	1.000
A:8oxoG	0.821	0.019	0.602	0.002	0.005	0.000	1.000	1.000	1.000
IR(3000Gy)	0.822	0.004	0.743	0.263	0.096	0.212	0.000	1.000	0.000
Hx(MPG)	0.613	0.542	0.001	0.510	0.905	0.836	0.166	0.000	1.000
NER	0.941	0.017	0.324	0.038	0.067	0.453	0.000	0.408	0.000

Baseline (row) - Post-RT (column) Pearson's correlation raw p value

	NHEJ	LP-BER	MMR	U:G	8oxoG:C	A:8oxoG	IR(3000Gy)	Hx(MPG)	NER
NHEJ	0.000	0.267	0.393	0.245	0.736	0.137	0.344	0.846	0.016
LP-BER	0.437	0.000	0.000	0.659	0.733	0.228	0.000	0.577	0.000
MMR	0.565	0.019	0.000	0.018	0.886	0.203	0.000	0.882	0.006
U:G	0.049	0.238	0.996	0.001	0.237	0.556	0.091	0.192	0.515
8oxoG:C	0.709	0.321	0.497	0.746	0.000	0.450	0.410	0.037	0.768
A:8oxoG	0.940	0.107	0.017	0.412	0.154	0.000	0.214	0.832	0.927
IR(3000Gy)	0.255	0.466	0.007	0.852	0.576	0.719	0.000	0.804	0.000
Hx(MPG)	0.546	0.331	0.002	0.006	0.337	0.170	0.409	0.000	0.229
NER	0.790	0.083	0.006	0.986	0.469	0.461	0.000	0.747	0.000

Supplementary table 4. Radiotherapy response in DNA repair capacity by follow-up months.

	NHEJ			LP-PBER			MMR			U:G		
**Post-RT month groups**	beta	SE	p_value	beta	SE	p_value	beta	SE	p_value	beta	SE	p_value
Within 1 month (N=18)	0.172	0.166	0.300	**0.474**	**0.202**	**0.021**	-0.025	0.146	0.864	**-0.527**	**0.153**	**0.001**
1-3 months (N=35)	0.146	0.115	0.207	0.176	0.142	0.218	-0.071	0.100	0.479	-0.171	0.104	0.102
3-6 months (N=25)	-0.055	0.134	0.684	0.099	0.165	0.550	0.011	0.117	0.926	-0.006	0.126	0.960
6-12 months (N=14)	-0.026	0.175	0.884	-0.041	0.214	0.850	0.013	0.153	0.931	-0.162	0.157	0.304
>12 months (N=5)	**-0.705**	**0.288**	**0.015**	-0.101	0.351	0.774	-0.197	0.253	0.438	0.145	0.257	0.573

	8oxoG:C			A:8oxoG			IR(3000Gy)			Hx(MPG)			NER		
**Post-RT month groups**	beta	SE	p_value	beta	SE	p_value	beta	SE	p_value	beta	SE	p_value	beta	SE	p_value
Within 1 month (N=18)	0.222	0.189	0.242	**-0.519**	**0.186**	**0.006**	0.094	0.197	0.635	-0.118	0.155	0.448	0.292	0.171	0.089
1-3 months (N=35)	0.151	0.120	0.212	-0.117	0.124	0.348	-0.084	0.133	0.530	0.058	0.103	0.576	-0.032	0.113	0.780
3-6 months (N=25)	0.109	0.149	0.469	-0.076	0.149	0.611	0.043	0.158	0.785	-0.155	0.126	0.220	0.007	0.136	0.957
6-12 months (N=14)	0.066	0.192	0.734	-0.161	0.190	0.398	-0.090	0.202	0.655	0.162	0.158	0.307	-0.112	0.174	0.521
>12 months (N=5)	-0.375	0.307	0.224	-0.102	0.314	0.746	-0.118	0.332	0.723	0.177	0.261	0.498	-0.242	0.288	0.403

Statistics are obtained from linear mixed effect models with the post-RT month groups as the main effect variables.

medRxiv preprint doi: https://doi.org/10.64898/2026.01.12.26343971; this version posted January 13, 2026. The copyright holder for this preprint (which was not certified by peer review) is the author/funder, who has granted medRxiv a license to display the preprint in perpetuity.
It is made available under a CC-BY 4.0 International license .

Supplementary table 5. Case control comparison for DNA repair capacity.

	**Case (N=18) vs Control (N=18)**			**Case (N=100) vs Control (N=18)***		
**DRC**	beta	SE	p_value	beta	SE	p_value
NHEJ	**0.850**	**0.277**	**0.006**	**0.810**	**0.195**	**<0.001**
LP-BER	0.388	0.289	0.196	0.302	0.228	0.188
MMR	-0.388	0.224	0.103	0.147	0.198	0.458
U:G	-0.094	0.166	0.581	-0.050	0.152	0.742
8oxoG:C	-0.535	0.338	0.125	-0.390	0.258	0.134
A:8oxoG	**-0.885**	**0.347**	**0.016**	**-1.162**	**0.220**	**<0.001**
IR(3000Gy)	0.135	0.341	0.694	0.088	0.217	0.686
Hx(MPG)	**-0.798**	**0.291**	**0.010**	-0.238	0.186	0.205
NER	0.259	0.232	0.281	0.157	0.198	0.428

	**Trend test by number of primary**			**Single primary vs control**			**Multi primaries vs control**		
**DRC**	beta	SE	p_value	beta	SE	p_value	beta	SE	p_value
NHEJ	**0.367**	**0.130**	**0.006**	**0.773**	**0.218**	**0.001**	**0.837**	**0.257**	**0.002**
LP-BER	0.027	0.144	0.851	0.328	0.246	0.186	0.132	0.291	0.653
MMR	-0.051	0.136	0.709	0.183	0.227	0.424	-0.05	0.273	0.855
U:G	0.067	0.093	0.470	-0.038	0.162	0.813	0.104	0.19	0.584
8oxoG:C	-0.092	0.162	0.571	-0.516	0.276	0.065	-0.298	0.325	0.362
A:8oxoG	**-0.532**	**0.141**	**<0.001**	-1.072	0.232	**<0.001**	-1.205	0.274	**<0.001**
IR(3000Gy)	0.078	0.144	0.588	0.087	0.246	0.725	0.159	0.293	0.59
Hx(MPG)	**-0.233**	**0.112**	**0.041**	-0.205	0.191	0.287	**-0.461**	**0.229**	**0.047**
NER	0.079	0.127	0.537	0.184	0.216	0.397	0.181	0.258	0.486

* adjusted for age and sex

Statistics are obtained from linear regression models.

A

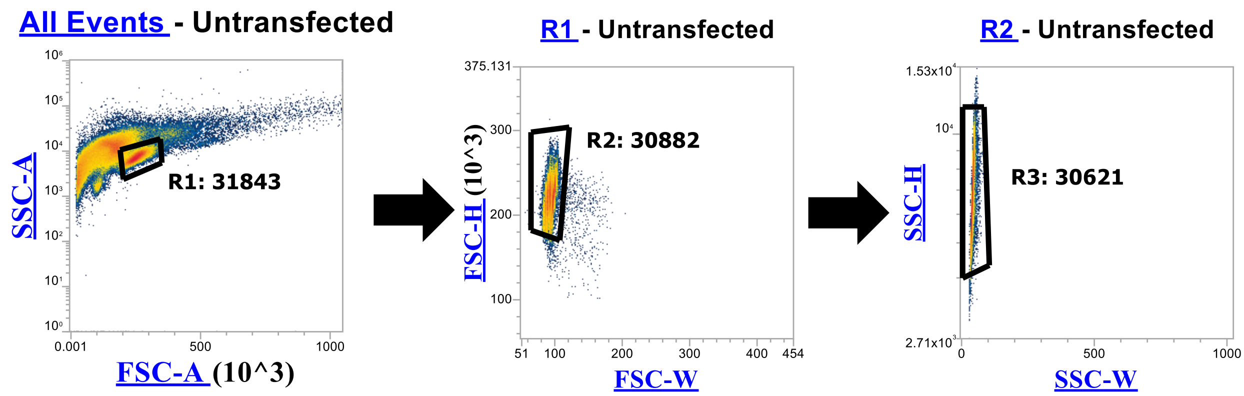

B

Experiment: **Group E_BroadbandCTRL_Sneh_13th Aug 2024**
Group: **E8**
Sample: **Untransfected**
Time Recorded: **19:10:49**

Name	Gate	X Parameter	Y Parameter	Count	%Gated	X Mean
All Events	All Events	N/A	N/A	117,367	100.000	N/A
R1	R1	FSC-A	SSC-A	31,843	27.131	251,825
R2	R2	FSC-W	FSC-H	30,882	96.982	94
R3	R3	SSC-W	SSC-H	30,621	99.155	42
BFP positive	BFP positive	VL1-H	YL1-H	0	0.000	N/A
GFP positive	GFP positive	BL1-H	VL1-H	0	0.000	N/A
mOrange positive	mOrange positive	YL1-H	YL3-H	0	0.000	N/A
mPlum positive	mPlum positive	YL3-H	YL1-H	0	0.000	N/A

Supp Figure 1

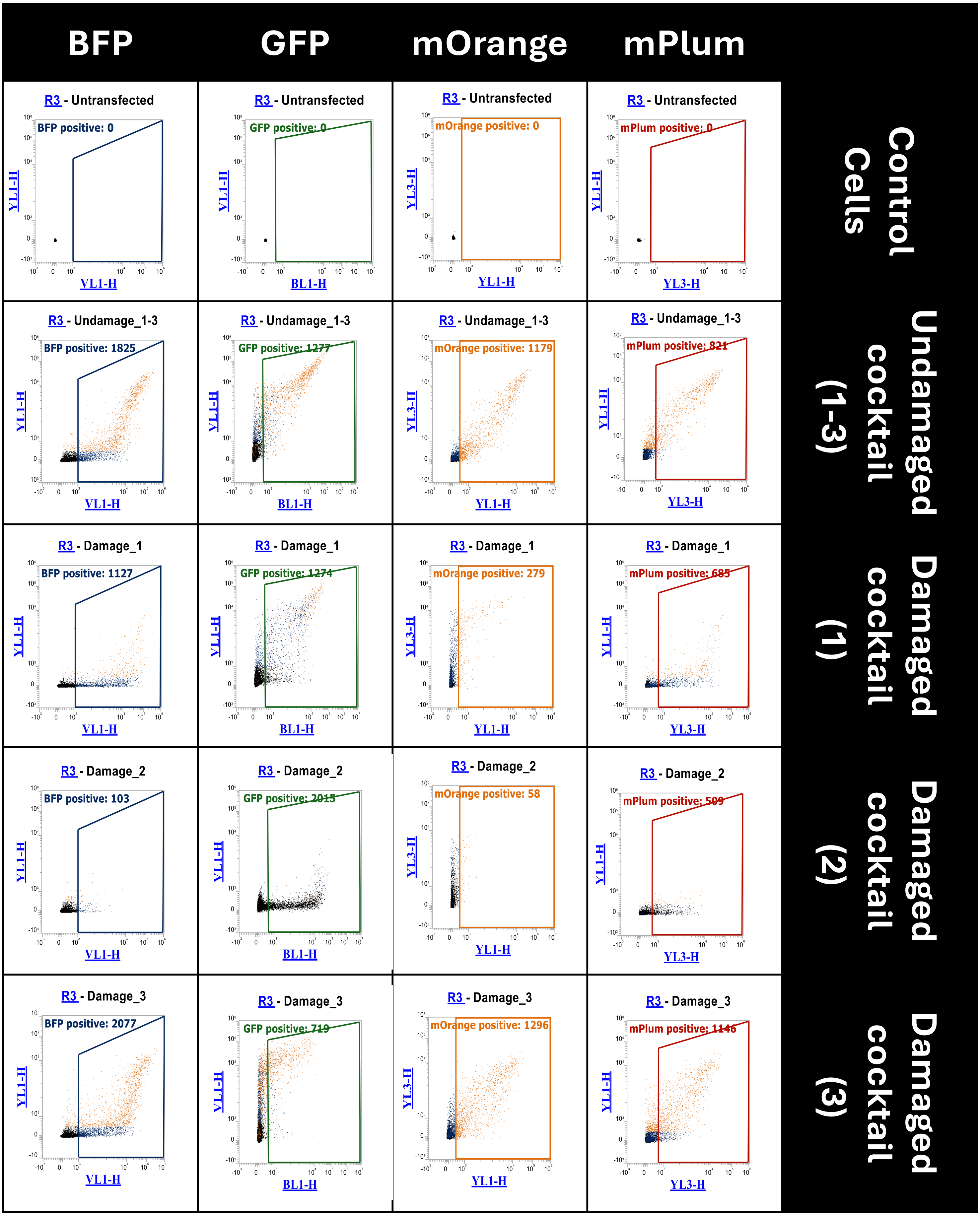

Supp Figure 2

A

B

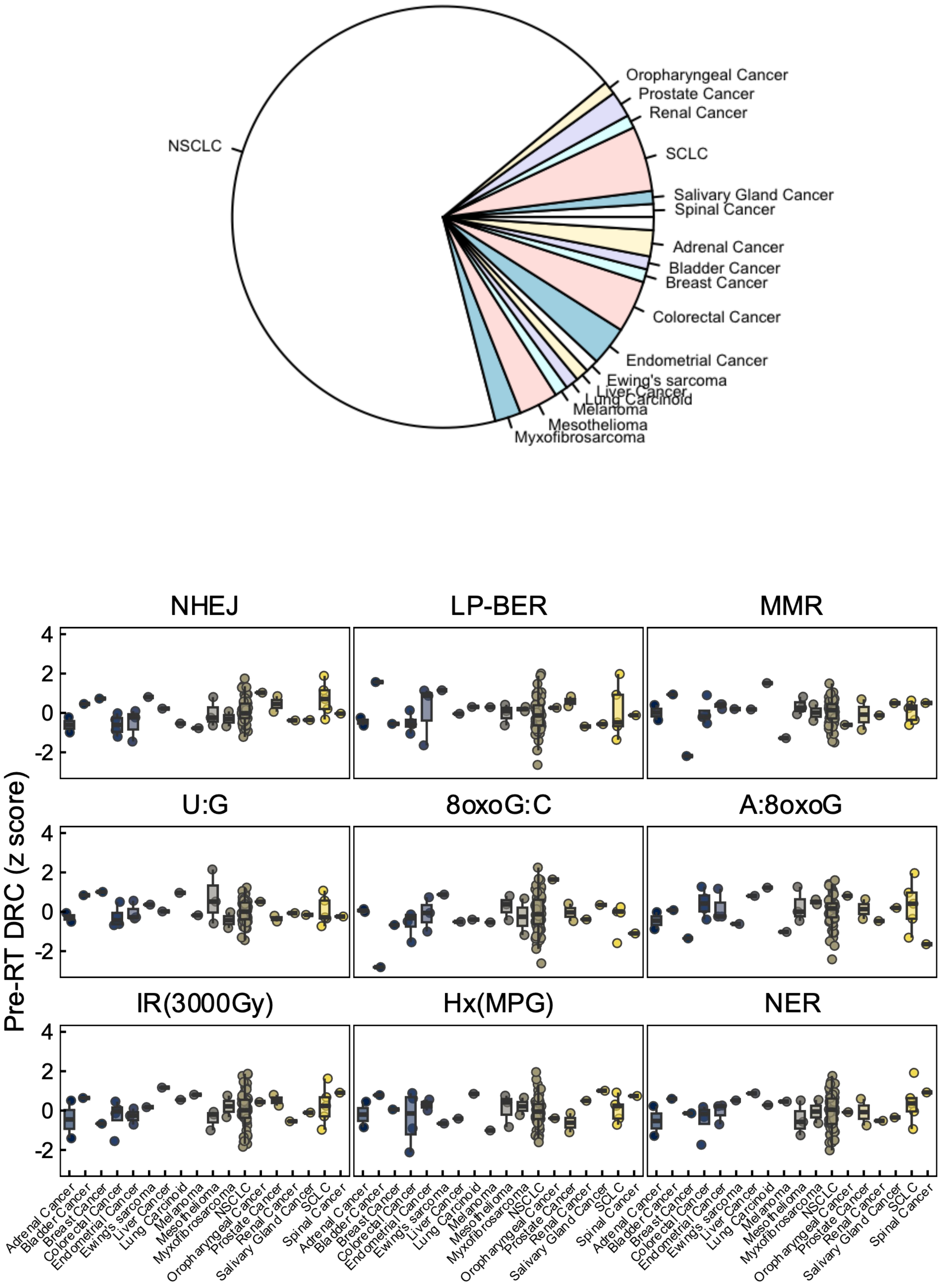

Supp Figure 3

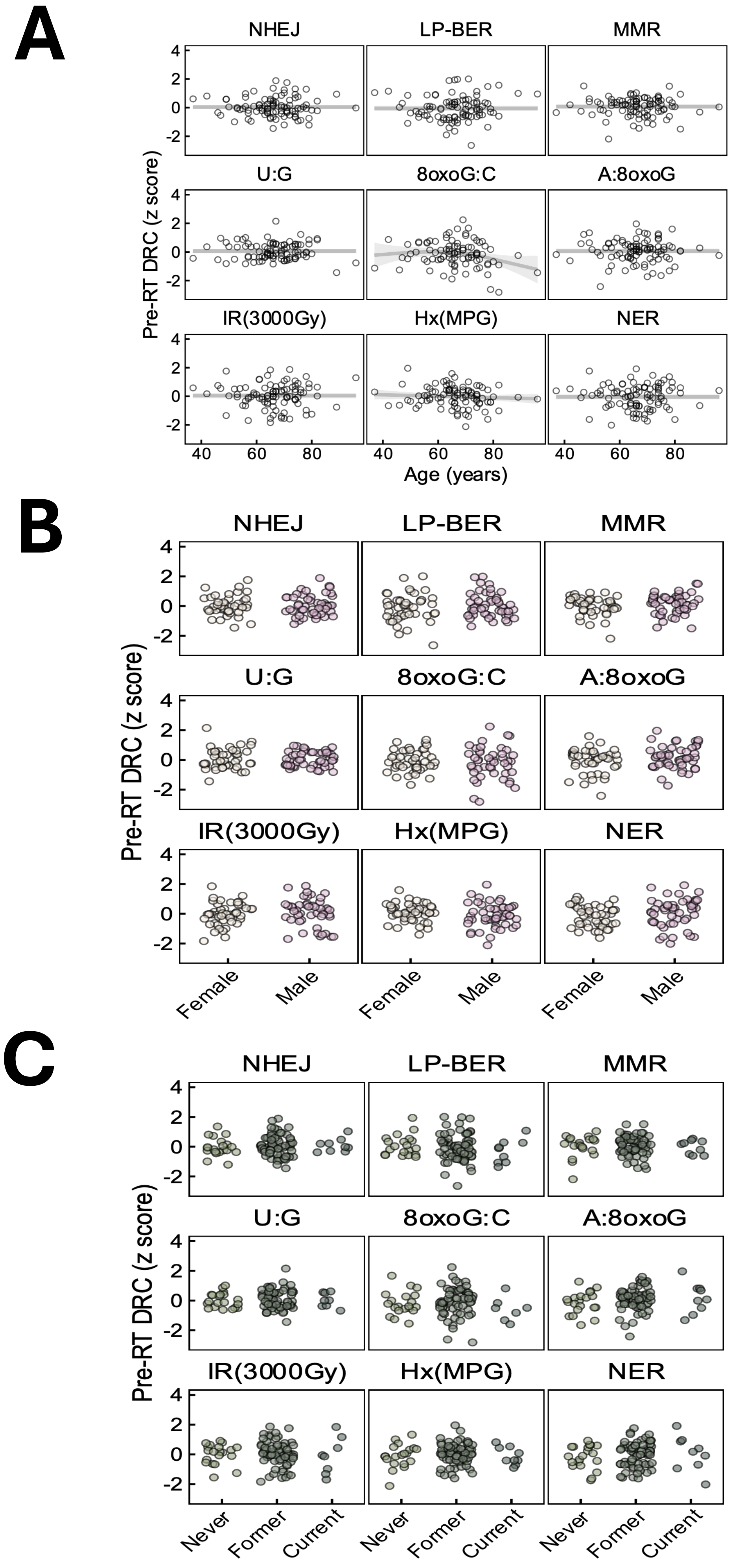

Supp Figure 4

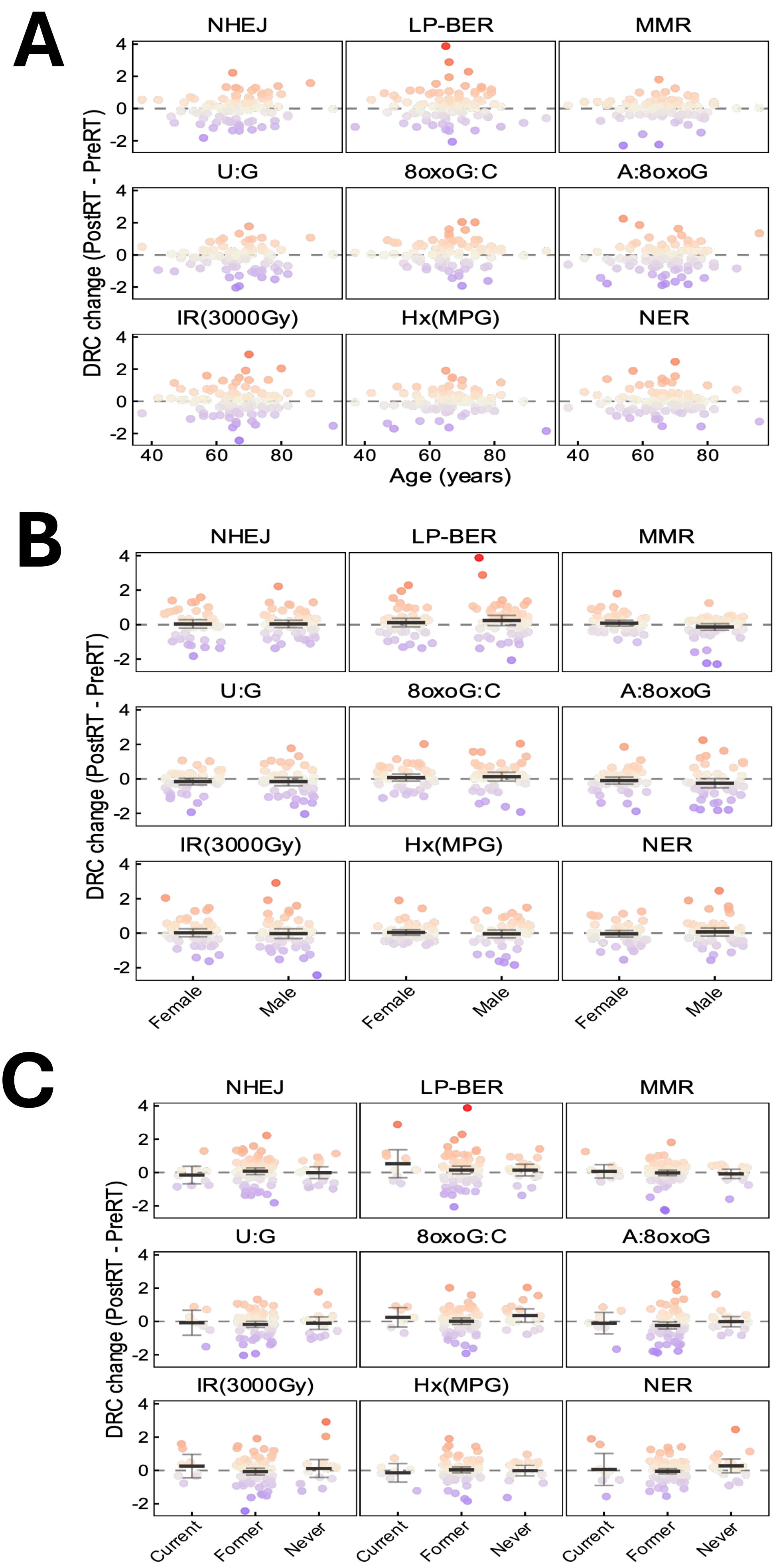

Supp Figure 5

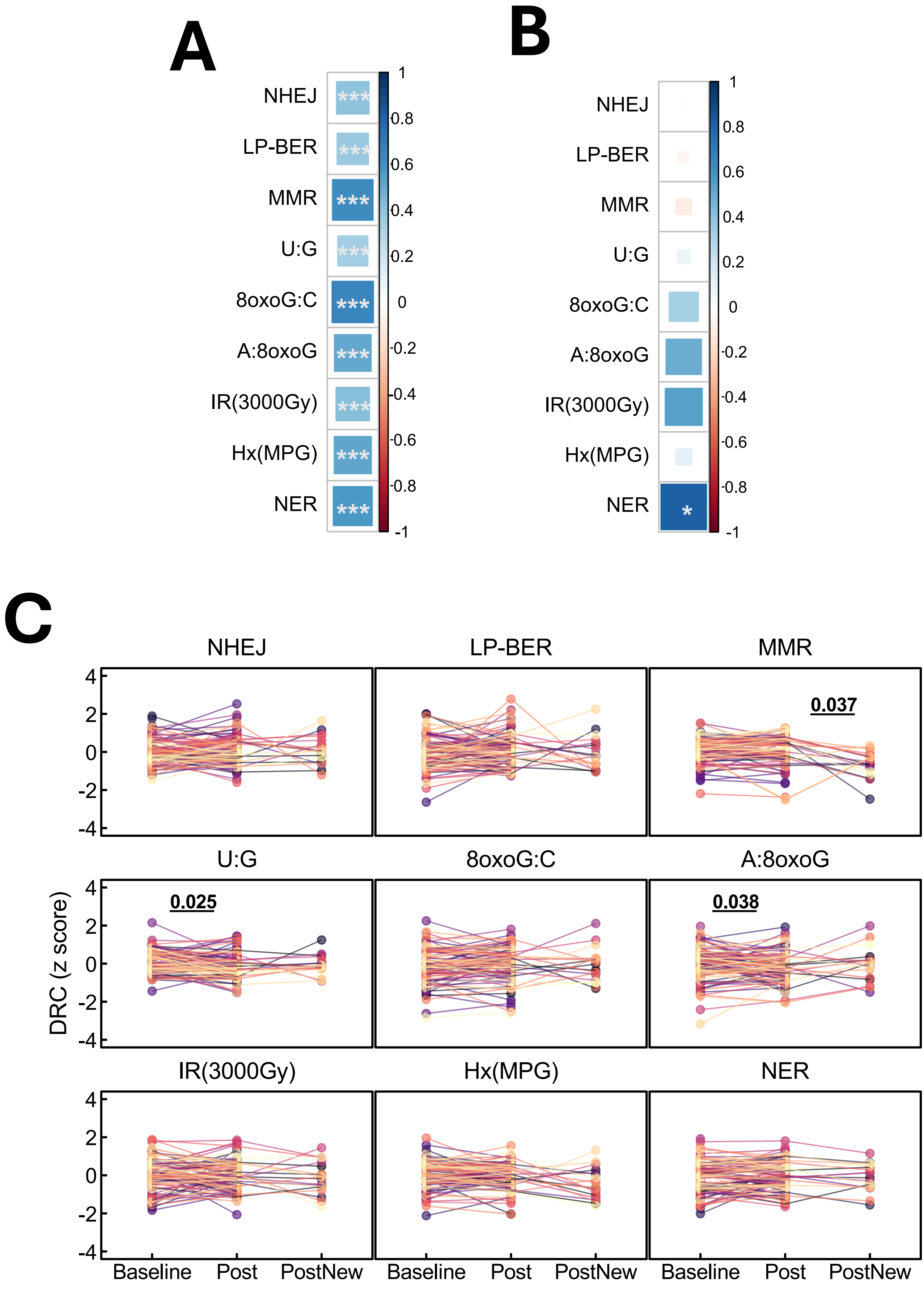

Supp Figure 6

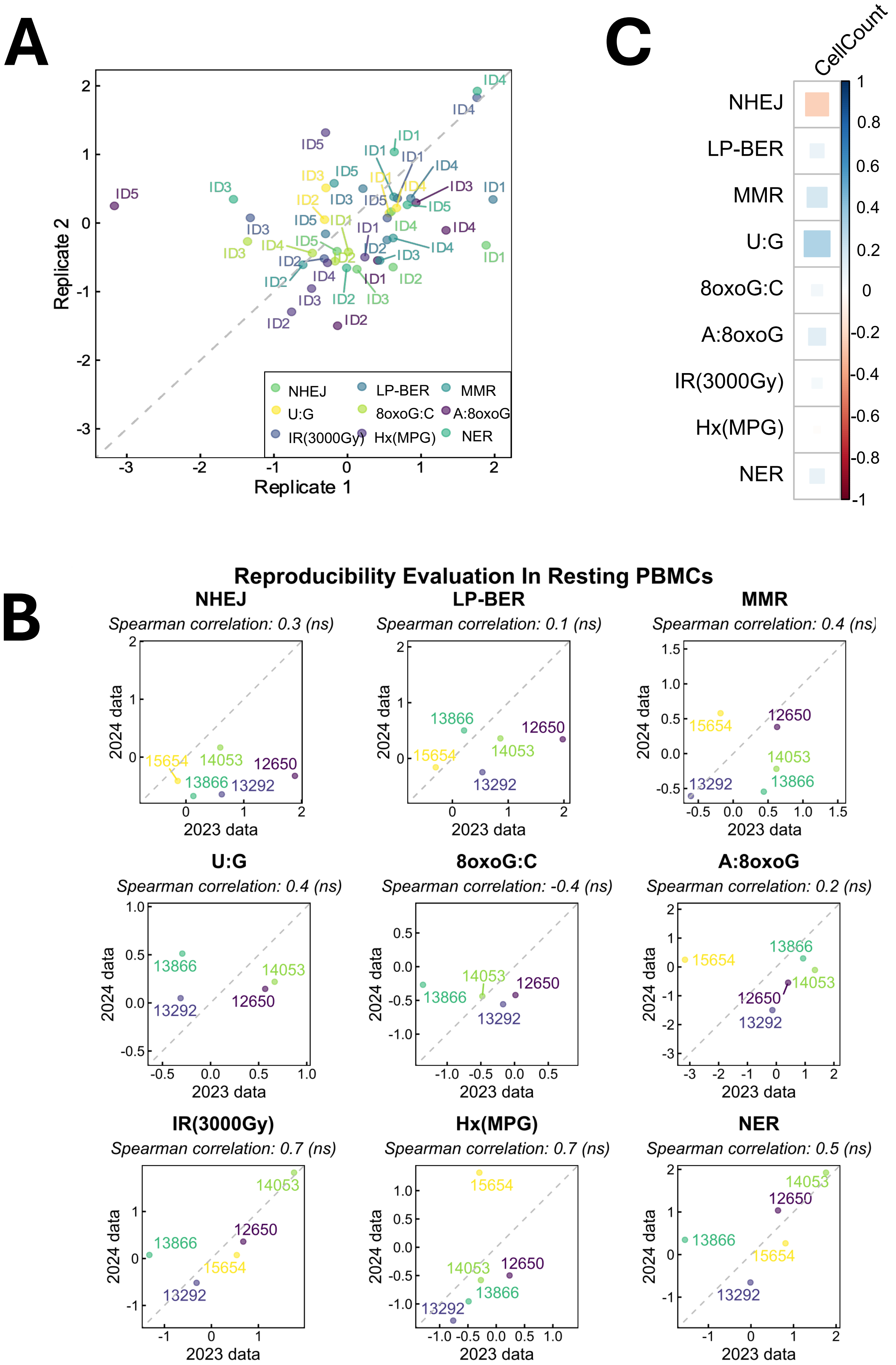

Supp Figure 7
